# HPV-Related Oropharyngeal Cancer and Biomarkers Based on Epigenetics and Microbiome Profile

**DOI:** 10.3389/fcell.2020.625330

**Published:** 2021-01-14

**Authors:** Spyridon Gougousis, Evangelia Mouchtaropoulou, Ioanna Besli, Paraskevas Vrochidis, Ioannis Skoumpas, Ioannis Constantinidis

**Affiliations:** ^1^GH “G. Papanikolaou,” ENT, Head and Neck Department, Thessaloniki, Greece; ^2^Center for Research and Technology–Hellas, Institute of Applied Biosciences, Thessaloniki, Greece; ^3^ENT Department, GH Goumenisa, Kilkis, Greece; ^4^1st Department of Otorhinolaryngology, Aristotle University of Thessaloniki, Thessaloniki, Greece

**Keywords:** head and neck cancer, HPV, methylation, miRNA, microbiome, biomarkers

## Abstract

H uman papillomavirus (HPV) is considered the main cause of the increasing incidence rates of oropharyngeal squamous cell carcinoma (OPSCC), and soon, the global burden of HPV-related OPSCC is predicted to exceed that of cervical cancer. Moreover, a different molecular profile for HPV-related OPSCC has been described, opening new promising targeted therapies and immunotherapy approaches. Epigenetic and microbiome-based exploration of biomarkers has gained growing interest with a view to the primary oropharyngeal cancer (OPC) screening. Understanding the role of the epigenetic mechanism and the changes that occur during pathogenesis shows appreciable progress in recent years. The different methylation status of DNA and miRNAs demonstrates the value of possible biomarkers discriminating even in different stages of dysplasia. Through whole-genome bisulfite sequencing, differentially methylated regions (DMRs) hold the key to recover missing information. O n the other hand, the microbiota investigation signifies a new biomarker approach for the evaluation of OPC. Along with known cofactors playing a major role in microbiota differentiation, HPV-related cases must be explored further for better understanding. The dynamic approach of the shotgun metagenomic sequencing will robustly fill the gap especially in species/strain level and consequently to biomarker detection. The constantly growing incidence of HPV-related OPC should lead us in further investigation and understanding of the unique features of the disease, more accurate diagnostic methods, along with the development and implementation of new, targeted therapies. This paper comprehensively reviews the significance of biomarkers based on epigenetics and microbiome profile in the accuracy of the diagnosis of the HPV-related cancer in the oropharynx.

## Introduction

### Epidemiology

Head and neck squamous cell carcinoma (HNSCC) is the eighth most common cancer worldwide (Stein et al., 2015). H uman papillomavirus (HPV)-related oropharyngeal squamous cell carcinoma (HPV-OPSCC) accounts for 25–30% of all HNSCC total cases (Tanaka and Alawi, 2018). HNSCC displays different characteristics in clinical symptoms, epidemiology, and treatment (surgical and pharmaceutical) because of the head and neck anatomical subsites and of the HPV presence in some of these cancers (Pan et al., 2018). HPV-OPSCC patients are usually described as white men, about 60 years old, little tobacco exposure, higher socioeconomic status, increased sexual behavior usually with same-sex contact, and earlier age at sexual debut. Among white people, about 1.8 women and 9.4 men per 100,000 were diagnosed with HPV-associated oropharyngeal cancer (OPC) (Centers for Disease Control and Prevention, 2020b). Predictions of the global percentage of HPV-OPSCC cases are about to transcend cervical cancer soon. HPV was first discovered by Jabłoñska and Gerard Orth in 1978 where they demonstrated that the virus was able to infect basal keratinocytes in the skin or mucosal membranes (Human Papillomaviruses, 2006). In 1983, Syrjänen (2005) was the first to describe the link between HPV infection and HNSCC. Later on, the explicit aspect of HPV-related cancers in the cervix was confirmed by Harald zur Hausen (Nobel Prize in Medicine 2008) (Pytynia et al., 2014).

However, difficulties in distinguishing the type of cancers arise from the oral cavity and the cancers from the oropharynx. Today, it is well established that HPV infection is a significant risk factor for the development of OPSCC.

Most importantly, HPV is the causal agent, about 70% (Centers for Disease Control and Prevention, 2020a), in a subset of OPSCCs, and the rising number of HPV-positive OPSCC patients has led to novel considerations regarding the diagnosis and therapeutic management of these patients.

### Diagnosis

HPV detection in OPCs is based on the concomitant assessment of p16 expression by immunohistochemistry (IHC) and the presence of the viral DNA by polymerase chain reaction (PCR)-based approaches. Interestingly, the association between p16 expression and HPV positivity does not exist among the non-OPCs. Although the assessment of p16 expression is routinely used along with the diagnosis of HPV infection, the gold standard remains the detection of HPV E6 and E7 messenger RNA (mRNA) expression *via* quantitative reverse transcription-PCR (qRT-PCR). This is indicative of a transcriptionally active virus within tumor cells (Ndiaye et al., 2014). Different technical methods are used to detect the existence of the HPV, such as *in situ* hybridization, Southern hybridization, and differential techniques to collect tumor tissues and samples. Hence, a substantial diversity concerning the percentage of HPV-related cancer exists. The use of saliva-based assays has been proposed as a simple and rapid test for detecting HPV-HNSCC, though it remains a subject for validation (Wasserman et al., 2017). For an accurate diagnosis of HPV-OPSCC, the importance of a patient’s history and physical examination, along with appropriate imaging, cannot be overemphasized (Lydiatt et al., 2017). In asymptomatic OPSCC patients, an enlarged lateral neck mass may be noticed, which often indicates metastasis to the cervical lymph node. Such occurs commonly in OPSCC due to the relatively late detection of the tumor. As the tumor progresses, symptoms such as dysphasia or tonsillar pain may present. Precancerous lesions may not be easily visualized or palpable during routine ear–nose–throat (ENT) examinations because of the preferred anatomic sites (tonsils, base of the tongue) of HPV-OPSCC. Ultimately, only a tissue biopsy provides histopathologic confirmation of the tumor. The HPV status of the tumor can be revealed by PCR or immunohistochemical staining, as previously discussed.

### Treatment

There are now data supporting the assumption that the HPV-OPSCC is a clinically distinct subset of HNSCC. HPV-OPSCC is associated with an overall better treatment outcome than non-HPV-OPSCC, with a higher survival rate and lower adverse effects being reported (Ang et al., 2010). In particular, HPV-OPSCC has shown a more supportive outcome when treated with radiation, either alone or with concomitant chemotherapy. In the current edition of the American Joint Committee on Cancer (AJCC) manual, HPV-OPSCCs are distinguished from non-HPV-OPSCCs. Clinical (cTNM) and pathologic TNM (pTNM) are used to stage p16-positive, high-risk HPV-OPSCC. cTNM is applied to all patients with p16-positive, high-risk HPV-positive OPSCC, whereas pTNM is used only for patients who undergo surgery for their cancers (Hoffmann and Tribius, 2019). An example of one of the striking changes is reflected in the reclassification of a p16-positive OPSCC cancer (2 cm tumor with two positive ipsilateral lymph nodes, T1N2M0) from stage IV in the previous AJCC manual to stage I. Although some challenges exist, including the potential difficulty of identifying the primary tumor site in advanced-stage OPSCC and a lack of case-controlled studies, better prediction of HPV-OPSCC prognosis is expected using both the clinical and the pathologic data sets (Wang et al., 2015). The molecular mechanisms underlying the differences in treatment response between HPV-positive and negative OPSCC remain unclear. A recent study suggests that p16 sensitizes HPV-positive tumor cells to ionizing radiation by inhibiting homologous recombination-mediated DNA repair (Dok et al., 2014). Although, the exact mechanism that increases the survival of HPV-positive OPSCC remains to be more fully elucidated.

Overall, laboratory detection methodologies of HPV-HNSCC patients do not apply same-scale sensitivity to all cases, resulting in misdiagnosis and inability to receive appropriate treatment. New insights will emerge through the discovery of biomarkers with high sensitivity and specificity and with dynamic prospects for tests of each anatomical site. Moreover, non-invasive screening must be further investigated through large-scale studies for early diagnoses, such as the cervix cases (Agorastos et al., 2019).

## Epigenetics

### DNA Methylation Status of Head and Neck Squamous Cell Carcinoma

DNA methylation is the epigenetic mechanism that is mainly involved in the physiological control of genome expression. The process by which DNA methylation functions to repress gene transcription from gene promoter regions is altered upon the development of cancer. Factors implicated in changes and inducing hypermethylation or hypomethylation pertain to alteration of the activity of DNA methyltransferases (DNMTs), inflammation, and viral infection (Toyota and Yamamoto, 2011). The role of hypermethylation is comprehensible in different types of cancer, whereas hypomethylation, acknowledged as being associated with repeated DNA sequences (Ehrlich, 2009), remains relatively less explored. Findings in a study in B cell chronic lymphocytic leukemia (B-CLL), emerges the interactive role of hypermethylation and hypomethylation status (Kushwaha et al., 2016). When it comes to HPV-related cancer, hypermethylation has been covered with sizing studies initially focusing on DNA methylation of either the virus or the host.

The hypermethylated promoter of tumor suppressor genes is considered to initiate carcinogenesis and to affect all cellular pathways with a tumor type-specific profile. Therefore, tumor suppressor genes represent the primary choice for biomarker exploration. *RASSF1A*, *TIMP3*, and *PCQAP/MED15* were presented as a four-panel approach for the earliest possible identification of the incidence of oral cancer (OC)/OPC (Liyanage et al., 2019). Remarkably, the *de novo* methylation of the promoter CpG islands of the *RASSF1A* gene triggers the initiation of OC (Ovchinnikov et al., 2012; Wen et al., 2018). The *PITX2* gene, which regulates the cell cycle, has been found to be hypermethylated in HNSCC samples and highly correlated with the HPV positivity. Additionally, oropharynx and oropharyngeal tumors presented higher levels of methylation compared to other tumor sites (Sailer et al., 2017). The *IDO1* gene encodes indoleamine 2,3-dioxygenase, whose increased expression has been associated with viral and bacterial infections as well as tumor pathogenesis. In HPV-associated tumors (HNSCC), increased expression of the IDO1 gene was found in conjunction with significantly lower DNA methylation in the promoter flank region. The research results were verified by further investigations of the regulator interferon γ (IFNγ) and the expression levels of all genes involved in the pathway [interferon gamma (IFNG), signal tranducer and activator of transcription (STAT)1, STAT2, Janus kinase (JAK)2, and IFN regulator factor (IRF)9] were positively correlated (Sailer et al., 2019). The sal-like 3 protein (SALL3) binds to DNA methyltransferase 3 alpha (DNMT3A), and the silencing of this gene is involved in the regulation of cell growth (Shikauchi et al., 2009). In the case of HNSCC, *SALL3* hypermethylation was associated with the expression of two of the three 10–11 translocation family enzymes (TET1, TET2) and DNMT3A methyltransferase, supporting the hypothesis that the *SALL3* gene may play a role in the tumorigenesis and may serve as an important biomarker (Misawa et al., 2017). *SALL2* is also suggested as a biomarker in the clinical risk assessment, significantly correlated with *SALL1* and *SALL3* methylation status but not with HPV infection (Imai et al., 2019). Finally, potential prognostic properties of *SALL1* indicate the power of discrimination in tumor stages T1 and T2 in HNSCC (Misawa et al., 2018). Complementary studies show evidence of oral dysplasia and OC of the hypermethylated genes *ZNF582, SOX1*, and *PAX1* (Cheng et al., 2016; Huang et al., 2017; Tang et al., 2019). A well-studied gene, the tumor suppressor *EPB41L3*, which inhibits cell proliferation and promotes apoptosis, in combination with early and late genes of high-risk HPV subtypes (hrHPV), has arisen as a promising biomarker panel for cervical cancer (Rogeri et al., 2018; Hernández-López et al., 2019; Kelly et al., 2019). For the first time, this panel triaged on OPC cases with evidence of the biomarker utility (Giuliano et al., 2020).

Although aberrant DNA methylation occurs mainly in promoter regions, CpG island shores and body regions provide valuable information about interactions and methylation profiles (Irizarry et al., 2009). A genome-wide analysis study found that 60% of differentially methylated genes (DMGs) were hypomethylated, and gene expression was more likely to be affected by hypomethylated DMRs. Namely, the genes *NCAN, NRXN1*, and *COL19A1*, involved in the organization of the extracellular matrix, and *SYCP2*, *RPA2*, and *SMC1B*, involved in the structural maintenance of chromosomes during mitosis/meiosis, were found to be hypomethylated and overexpressed in HPV-positive cancers. Moreover, the average methylation levels of significant hyper- (top 25) or hypo- (top 25) DMRs are able to separate HPV-positive from HPV-negative HNSCC specimens for each anatomical site (Esposti et al., 2017). Studies on related scales underline the importance of a novel approach by whole-genome sequencing and how predictive accuracy can vary individually (Ren et al., 2018; Das et al., 2019; Gašperov et al., 2020).

### MicroRNA Prospects in Head and Neck Squamous Cell Carcinoma

Attention has been focused recently on the small non-coding RNA molecules, miRNAs, which are known for RNA interference and posttranscriptional regulation of gene expression. miRNAs target either one gene or simultaneously different genes, thus involved in multiple cell signaling pathways. This epigenetic mechanism of miRNAs is also considered a modulator of histone modifications (Morales et al., 2017) by increasing their target activity when genes with their promoter regions are located in the active chromatin state regions (Tao et al., 2017). Their abnormal expression level is relentlessly studied in recent years, indicating the association in various human cancers (Anastasiadou et al., 2017, 2019; Marco et al., 2018). Some viruses also encode miRNAs (Anastasiadou et al., 2010; Rosato et al., 2012), demonstrating to regulate the host’s immune system (Iizasa et al., 2020). On the other hand, the epigenetic regulation of these molecules is covered to a small extent, although indications seem to be significant (Wang et al., 2017).

TP53 tobacco-associated mutations are frequent observations in head and neck tumors. Various miRNA signature associations and the evaluation of the TP53 mutation profile have highlighted their importance for clinical outcome and tumor in tissues (Ganci et al., 2013; Metheetrairut et al., 2019; Chari et al., 2020). Emphasizing the individual miRNAs, miR-145 is a p53-regulated gene that acts as a metastasis suppressor by targeting multiple genes in different types of cancer (Sachdeva and Mo, 2010; Leite et al., 2013). This role is also confirmed in laryngeal squamous cell carcinoma (LSCC) by the regulatory axis miR-145-5p/FSCN1 (Gao et al., 2019). Even more, this specific miR-145 examined on HPV(+) and HPV(−) HNSCC samples shows that HPV(+) tumor cases have a distinct miRNA profile of the miR-15a/miR-16/, miR-143/miR-145, and the miR-106-363 cluster (Lajer et al., 2012). miR-373 belongs to a cluster of four miRNAs located on chromosome 19q13 and has attracted research interest stated as an oncomir in esophageal cancer. The upregulation of the tumor suppressors mir-373 and mir-372 has been associated with proliferation, invasion, and metastasis in oropharyngeal samples, indicating a worse survival overexpression (Tu et al., 2015; Zhang et al., 2019). Moreover, a study focusing on miR-373-3p in tumor tissues showed that expression levels may be regulated by the hypomethylated miR-373-3p promoter that mediates the developmental process of esophageal squamous cell carcinoma (ESCC) (Wang L. et al., 2019). Contrarily, miR-148a-3p targeting the long non-coding RNA H19 (lncRNA H19) and the DNA methyltransferase enzyme DNMT1 shows to suppress migration and invasion of cancer cells and may have prognostic value in the future (Wu et al., 2016; Wang Y. et al., 2019).

Investigation of individual miRNA molecules may be of use for the benefit of diagnostic and prognostic value in HNSCC, but when it comes to a combination of miRNAs, provides a more significant prediction in clinical outcomes. The diagnostic power of miR-383, miR-615, and miR-877 panel distinguishes patients with HNSCC from healthy donors, with high rates of a receiver operating characteristic (ROC) curve analysis (89.3% sensitivity and 98.9% specificity) (Liu et al., 2019). Given the unfavorable prognosis of HPV-negative cancer patients strengthens the need to find biomarkers through miRNA signatures for a more accurate diagnosis (Hess et al., 2019).

## Microbiome

The microbiome is one of the developing fields of study and evaluation in recent years not only as a single element in monitoring the normal flora of organisms but also coexistence between host and bacteria. Although great efforts have been made, little is known about the consideration of normal flora due to various factors that affect the microbial community such as age, gender, genetics, mode of birth (normal birth or cesarean section), and nutritional factors (Zapata and Quagliarello, 2015; Dominguez-Bello et al., 2019). Nevertheless, the host–bacteria symbiosis has proven to play an important role in metabolic functions (Chow et al., 2010), and any imbalance could lead to insulin resistance, inflammation, vascular and metabolic disorders (Pascale et al., 2018), and cancer pathogenesis (Chen et al., 2017; Stashenko et al., 2019).

The oral cavity has been characterized by Actinobacteria, Bacteroidetes, Chlamydiae, Chloroflexi, Euryarchaeota, Firmicutes, Fusobacteria, GN02, Proteobacteria, Spirochaetes, SR1, Synergistetes, Tenericutes, and TM7 bacteria taxa at the phylum level, according to the Human Oral Microbiome Database (HOMD). Factors such as smoking, betel quid chewing (Yu et al., 2017), and alcohol consumption (Fan et al., 2018) have been adequately associated with changes of oral flora, giving space for colonization of opportunistic pathogens. Additionally, poor oral hygiene leads to changes in the ratio of bacterial flora, triggering inflammatory oral diseases such as periodontitis and gingivitis (Kilian et al., 2016; Schulz et al., 2019).

Different bacterial profiles in oral squamous cell carcinoma (OSCC) point out that multiple cofactors are playing a crucial role. A comparative study showed that switched microbiota depends on different tissue sites (tumor sites and non-tumor sites). *Streptococcus* sp. oral taxon 058, *Peptostreptococcus stomatis*, *Streptococcus salivarius*, *Streptococcus gordonii*, *Gemella haemolysans*, *Gemella morbillorum*, *Johnsonella ignava*, and *Streptococcus parasanguinis* were highly associated with tumor site, whereas *Granulicatella adiacens* was prevalent at the non-tumor site (Pushalkar et al., 2012).

OPC and hypopharyngeal (HP) cancer are distinguishable through a differential microbiome profile. *Streptococcus anginosus* was only significantly elevated in saliva of OPC patients (Panda et al., 2020). Mutational changes can also variate the relative abundance of bacteria, such as Firmicutes and Bacteroidetes found to be different among groups concerning different mutants (Yang et al., 2018). Considering that HPV promotes carcinogenesis in some cases, studies that included HPV-positive samples in conjunction with oral flora assessment showed different results. A small pilot study found that patients with OC/OPC differed significantly from healthy controls. Interestingly, in the case of HPV-positive samples was demonstrated as a “normal” microbiome profile (Wolf et al., 2017). In contrast, an HPV-positive correlation was found between the genera *Haemophilus* and *Gemella* in oral cavity cancer (OCC) and OPC. Additionally, *Actinomyces*, *Parvimonas*, *Selenomonas*, and *Prevotella* were more abundant in OCC compared to OPC (Lim et al., 2018). On the other hand, a case-control study pointed out that *Corynebacterium* and *Kingella* are associated with a decreased risk of OC (Hayes et al., 2018). At species level, it was found that *Streptococcus salivarius–Streptococcus vestibularis* are abundant in OSCC samples, and more importantly, analysis of subsets of these samples showed that species of the vaginal flora are abundant in saliva (*Lactobacillus gasseri/johnsonii* and *Lactobacillus vaginalis*) (Guerrero-Preston et al., 2017).

Associations between the microbial profile and cancer have so far been based mainly on the sequencing of bacterial 16S ribosomal RNA (rRNA) genes. It has been shown that significant differences in the analysis of whole bacterial genomes improve the characterization of microbial communities, even at the subtype/strain level (Ranjan et al., 2016; Brumfield et al., 2020).

## Discussion

Molecular biomarkers have become an imperative need for the better, immediate diagnosis and treatment of patients with OPC. The two main areas of research of epigenetics (Table 1) and microbiome profile (Table 2) show excellent prospects. In the case of DNA methylation, several biomarkers have been proposed to differentiate even cancer stages. Regarding microbiome profiles, factors that play a crucial role in changes in flora have been established, and clinical studies have highlighted the importance of probiotic groups as therapy in dental diseases (Toiviainen et al., 2014; Alanzi et al., 2018). However, HPV-related cases are less explored compared to cervical microbiome flora samples. A multifaceted search for biomarkers would allow a better understanding of the molecular mechanisms of carcinogenesis by replacing targeted investigation. Whole-genome bisulfite and shotgun metagenome sequencing, which involves the random sequencing of all genomic content of a microbiome, will contribute to the achievement of this goal in combination with improved bioinformatic analysis. Finally, the synchronous inclusion of the two investigational biomarker fields of interest could promote primary head and neck cancer screening.

**TABLE 1:** Epigenetic mechanisms related in HNSCC.

				Methylation status		
	
Author	Tissue	Samples	HPV-related	Hypermethylation	Hypomethylation	Results
**DNA methylation**						
Liyanage et al. (2019)	Saliva	OC/OPC/Controls	Yes	p16INK4a, RASSF1A, TIMP3, PCQAP/MED15	–	Discrimination and early detection of OC/OPC
Wen et al. (2018)	Oral rinse/Tissue/Blood	OSCC/Controls	No	PASSF1A, RARb, CDH1 (meta-analysis)	–	Association with the oral cancer risk
Ovchinnikov et al. (2012)	Saliva	HNSCC/Controls	No	RASSF1A, DAPK1, p16INK4a	–	Prediction of incidence risk in HNSCC
Sailer et al. (2017)	Tissue	HNSCC/Controls	Yes	PITX2	–	Prognostic biomarker in HNSCC
Sailer et al. (2019)	Tissue, Cell lines	HNSCC/Controls	Yes	IDO1	–	Potential biomarker for prediction of response to IDO1 immune checkpoint inhibitors
Misawa et al. (2017)	Tissue	HNSCC/Controls	Yes	SALL3	–	Potential biomarker in HNSCC
Imai et al. (2019)	Tissue	HNSCC/Controls	Yes	SALL2	–	Important clinical risk assessment
Misawa et al. (2018)	Tissue, Cell lines	HNSCC/Controls	Yes	TET1,TET2,TET3	–	TET3 methylation independently associated with aggressive tumor
Cheng et al. (2016)	Oral scrapings	OPMD/OSCC/Controls	No	ZNF582, PAX1	–	Detection of oral dysplasia and oral cancer and prediction of oral cancer recurrence
Huang et al. (2017)	Tissue (tumor/paracancerous)	ESCC/Controls	No	ZNF582, PAX1	–	Distinguishing ESCC tumor tissues from non-tumor tissues
Tang et al. (2019)	Tissue	ESCC/Controls	No	PAX1, SOX1, ZNF582	–	Promising biomarker for ESCC screening and diagnosis
Giuliano et al. (2020)	Oral gargles	OPC/Controls	Yes	EPB41L3	–	Possible utility in identifying OPC early
Esposti et al. (2017)	Tissue	HNSCC	Yes	CDH18, CTNND2 (methylome analyses)	NCAN, NRXN1, COL19A1, SYCP2, RPA2, SMC1B (methylome analyses)	Novel epigenetic signature of HPV infection in HNSCCs independent of the anatomic site
Das et al. (2019)	Tissue	OSCC-GB/Controls	No	TET1 (methylome analyses)	CD274, CD80, DNMT3B (methylome analyses)	Indication of novel therapeutic targets, including immunotherapeutic, for treatment of OSCC-GB
Ren et al. (2018)	Tissue	OPSCC/Controls	Yes	OR6S1, KCNA3,EMBP1, CCDC181, DPP4,ITGA4,BEND4, ELMO1,SFMBT2,C1QL3, MIR129–2,NID2, HOXB4,ZNF439,ZNF93, VSTM2B, ZNF137P, ZNF773 (methylome analyses)	–	20 highly specific DMRs in HPV- related OPSCC, with potential application to molecular-based detection tests
Gašperov et al. (2020)	Tissue	Oral lesions/HNSCC/Controls	Yes	RAD51B, BARX2, SLC5A10/FAM83G, NINL NSMCE2, PGAP2, INO80C, IL34, ZNF516, GFOD2, PARD3, MCEE, POLM, ASPG, TBC1D2 (Promoters in HNSCC Tissue Compared to Oral lesions-methylome analyses)	ART4, EPB41L3, ESRRG, ENPP1, GNG7, PAPSS2, NGEF, HIPK4, GPR158, GSG1L, SMPD3, GDF2, RERE, CDH13, HS3ST4 (Promoters in HNSCC Tissue Compared to Oral lesions-methylome analyses)	Potential biomarkers for early diagnostics of HNSCC and premalignant oral lesions
**miRNAs**						
Gao et al. (2019)	Tissue	HNSCC/LSCC/Controls	No	miR-145-5p	Promoter hypermethylation	miR-145-5p and FSCN1 are important potential prognostic markers and therapeutic targets for LSCC
Lajer et al. (2012)	Tissue	HNSCC/TSCC/OSCC/PSCC/Control	Yes	miR-15a/miR-16/miR195/miR-497 family, miR-143/miR-145 and the miR-106-363 cluster	–	New knowledge to known pathogenic pathways of HPV and substantiates the oncogenic role of HPV in subsets of HNSCCs
Tu et al. (2015)	Tissue/Cell lines	OSCC/Control	No	miR-372, miR-373	–	Overexpression of miR-372 and miR-373 indicates worse survival in OSCC
Zhang et al. (2019)	Tissue/Cell lines	OSCC/Control	No	miR-373	–	MiR-373/SPOP potential therapeutic target for OSCC
Wang L. et al. (2019)	Tissue/Cell lines	ESCC/Control	No	miR-371a-5p, miR- 371b-5p, miR-372-3p, miR-373-3p	miR-373-3p promoter hypomethylation	DNA epigenetic modification in the miR-373-3p promoter region and the Hippo and p53 signaling pathways play important roles during the miR-373-3p mediating ESCC development process
Wu et al. (2016)	Tissue	LSCC/Control	No	lncRNA H19/miR-148a-3p/DNMT1	Promoter hypomethylation	lncRNA H19 promoted LSCC progression *via* miR-148a-3p and DNMT1
Wang Y. et al. (2019)	Tissue/Cell lines	ESCC/Control	No	miR-148a-3p	–	miR-148a-3p, by targeting DNMT1, likely regulates cell proliferation and invasion in esophageal cancer. Might also be used prognostically in esophageal cancer and serve as a therapeutic target in the future
Liu et al. (2019)	Tissue	HNSCC/Control	Yes	128 miRNAs as significantly differentially expressed in HNSCC tissue compared with the normal samples	–	hsa-miR-383, hsa-miR-615, and hsa-miR-877 may serve as an excellent diagnostic biomarker for HNSCC, and potential prognostic significance for HNSCC patients
Hess et al. (2019)	Tissue	HNSCC/DKTK-ROG/LMU-KKG (radiochemotherapy received)	Yes (negative)	hsa- let-7g-3p, hsa-miR-6508-5p, hsa-miR-210-5p, hsa-miR-4306, and hsa-miR-7161-3p	–	The five-miRNA signature is a strong and independent prognostic factor for disease recurrence and survival of patients with HPV-negative HNSCC

*DKTK-ROG, German Consortium for Translational Cancer Research-Radiation Oncology Group; ESCC, esophageal squamous cell carcinoma; HNSCC, head and neck squamous cell carcinoma; LMU-KKG, Ludwig- Maximilians-University of Munich-Clinical Cooperation Group; LSCC, laryngeal squamous cell carcinoma; OC, oral cancer; OPC, oropharyngeal cancer; OPMD, oral potentially malignant disorders; OSCC, oral squamous cell carcinoma; OSCC-GB, Gingivo-buccal oral squamous cell carcinoma; PSCC, pharyngeal squamous cell carcinoma; TSCC, tonsillar squamous cell carcinoma.*

**TABLE 2:** Bacteria abundance in HNSCC.

Author	Tissue	Samples	HPV-related	Method	16S rRNA region	Bacteria abudance (Tumor)	Bacteria abudance (non-tumor)	Results
**Microbiome profile**								
Pushalkar et al. (2012)	Tissue	OSCC/Control	No	Culture-independent, cloning, sequencing	V4-V5	*Streptococcus* sp. oral taxon 058, *Peptostreptococcus stomatis*, *Streptococcus salivarius, Streptococcus gordonii, Gemella haemolysans, Gemella morbillorum, Johnsonella ignava*, and *Streptococcus parasanguinis* I	*Granulicatella adiacens*	Bacterial diversity in the oral mucosal tissues from non-tumor and tumor sites of OSCC subjects
Panda et al. (2020)	Saliva	OPC/HPC/Control		Next-generation sequencing	V3–V4	*Haemophilus parainfluenzae, Haemophilus influenzae* and *Prevotella copri* and lower abundance of *Rothia mucilaginosa*, *Aggregatibacter segnis, Veillonella dispar, Prevotella nan- ceiensis, Rothia aeria, Capnocytophaga ochracea, Neisseria bacilliformis, Prevotella nigrescens*, and *Selenomonas* noxia in OP and HP cancer patients./*Streptococcus anginosus* in OP cancer	–	Possible non-invasive diagnostic biomarker for OP and HP cancer patients. *Streptococcus anginosus* may be considered as a non-invasive diagnostic biomarker for OP cancer patients only
Yang et al. (2018)	Saliva	OSCC (3 TP53 mutational groups)MSC1/2/3	No	Next-generation sequencing	V4	Firmicutes (MSC2), Bacteroidetes and Synergistetes (≠ MSC2 <*x*< MSC3), *Seleno- monas* and *Rothia* (≠ MSC1<*xMSC*2 < xMSC3), *Capnocytophaga* (MSC3)	–	Oral microbiota is compositionally and functionally associated with the mutational changes in oral cancer
Wolf et al. (2017)	Saliva	OCC/OPC/Control	Yes	Next-generation sequencing	–	*Actinomyces* (Actinobacteria), *Schwartzia* (Firmicutes), *Treponema* (Spirochaetes), and *Selenomonas* (Firmicutes)	Bacteroidetes, Proteobacteria, Firmicutes	Evidence that differences in microbial abundance and diversity might inform disease status in SCC patients
**Microbiome profile**								
Lim et al. (2018)	Oral rinse	OCC/OPC/Control	Yes	Next-generation sequencing	V6–V8	*Rothia, Haemophilus*, *Corynebacterium*, *Paludibacter, Porphyromonas, Oribacterium*, and *Capnocytophaga* discriminate OCC and OPC patients from age-matched normal healthy individuals/*Haemophilus* and *Gemella* positive correlation with HPV infection	–	Oral microbiome prediction of the presence of OCC and OPC with sensitivity and specificity of 100 and 90%, respectively
Hayes et al. (2018)	Mouth washes	HNSCC/Control	Yes	Pyrosequencing	V3–V4	*Corynebacterium* (order Corynebacteriales), *Kingella* (order Neisseriales), *Neisseria* (order Neisseriales), *Abiotrophia* (order Lactobacillales), *Capnocytophaga* (order Flavobacteriales) and species *Kingella* dentificans and *Streptococcus sanguinis* were associated with reduced risk for larynx cancer	–	Greater oral abundance of commensal *Corynebacterium* and *Kingella* is associated with decreased risk of HNSCC
Guerrero-Preston et al. (2017)	Saliva	HNSCC/Control	Yes	Next-generation sequencing (454)	V3–V5	*Fusobacterium nucleatum* (600x higher), *Lactobacillus gasseri/johnsonii* (710x higher), *Lactobacillus vaginalis* (52x higher), *Streptococcus salivarius*: *Streptococcus vestibularis*	–	*Fusobacterium nucleatum, Lactobacillus gasseri/johnsonii*, and *Lactobacillus vaginalis* associated to oral and oropharyngeal cancer in saliva from HPV positive and HPV negative patients treated with surgery and chemoradiation

*HNSCC, head and neck squamous cell carcinoma; HPC, hypopharyngeal cancer; MSC, mutational signature cluster; OCC, oral cavity cancer; OPC, oropharyngeal cancer; OSCC, oral squamous cell carcinoma.*

## Author Contributions

SG and EM wrote the manuscript according to each one’s relevant cognitive object. IB, PV, and IS assessed the literature. IC conceived the original idea and supervised the manuscript. All authors contributed to the article and approved the submitted version.

## Conflict of Interest

The authors declare that the research was conducted in the absence of any commercial or financial relationships that could be construed as a potential conflict of interest.
